# The contribution of functional cognition screening during acute illness hospitalization of older adults in predicting participation in daily life after discharge

**DOI:** 10.1186/s12877-022-03398-5

**Published:** 2022-09-12

**Authors:** Maya Arieli, Maayan Agmon, Efrat Gil, Rachel Kizony

**Affiliations:** 1 Department of Occupational Therapy, Faculty of Social Welfare & Health Sciences, University of Haifa, Mount Carmel, 31905 Haifa, Israel; 2 The Cheryl Spencer Department of Nursing, Faculty of Social Welfare and Health Science, University of Haifa, Haifa, Israel; 3 Geriatric Unit, Clalit Health Services, Haifa and West Galilee, Haifa, Israel; 4 Faculty of Medicine, Technion, Haifa, Haifa, Israel; 5 Department of Occupational Therapy, Sheba Medical Center, Tel Hashomer, Ramat-Gan, Israel

**Keywords:** Executive functions, Performance-based screening measures, Functional cognition, Participation, Acute hospitalization, Older adults

## Abstract

**Background:**

Cognitive assessment in acutely hospitalized older adults is mainly limited to neuropsychological screening measures of global cognition. Performance-based assessments of functional cognition better indicate functioning in real-life situations. However, their predictive validity has been less studied in acute hospital settings. The aim of this study was to explore the unique contribution of functional cognition screening during acute illness hospitalization in predicting participation of older adults one and three months after discharge beyond traditional neuropsychological measures.

**Methods:**

This prospective longitudinal study included 84 older adults ≥ 65 years hospitalized in internal medicine wards due to acute illness, followed by home visits at one month and telephone interviews at three months (*n* = 77). Participation in instrumental activities of daily living, social and leisure activities was measured by the Activity Card Sort. In-hospital factors included cognitive status (telephone version of the Mini-Mental State Examination, Color Trails Test), functional cognition screening (medication sorting task from the alternative Executive Function Performance Test), emotional status (Hospital Anxiety and Depression scale), functional decline during hospitalization (modified Barthel index), length of hospital stay, the severity of the acute illness, symptoms severity and comorbidities.

**Results:**

Functional cognition outperformed the neuropsychological measures in predicting participation declines in a sample of relatively high-functioning older adults. According to a hierarchical multiple linear regression analysis, the overall model explained 28.4% of the variance in participation after one month and 19.5% after three months. Age and gender explained 18.6% of the variance after one month and 13.5% after three months. The medication sorting task explained an additional 5.5% of the variance of participation after one month and 5.1% after three months, beyond age and gender. Length of stay and the Color Trails Test were not significant contributors to the change in participation.

**Conclusions:**

By incorporating functional cognition into acute settings, healthcare professionals would be able to better detect older adults with mild executive dysfunctions who are at risk for participation declines. Early identification of executive dysfunctions can improve continuity of care and planning of tailored post-discharge rehabilitation services, especially for high-functioning older adults, a mostly overlooked population in acute settings. The results support the use of functional cognition screening measure of medication management ability in acute settings.

## Background

Resumed participation in a wide range of life domains is the desired outcome after hospitalization due to acute illness. Participation in meaningful activities is central to healthy aging and its deterioration is associated with lower quality of life, depressive symptoms and, cognitive decline [1, 2]. However, most studies conducted during and after acute hospitalization examined predictors of decline in Basic Activities of Daily Living (BADL) such as personal hygiene and dressing [3, 4] while overlooking other domains of participation relevant to high-functioning older adults such as outdoor recreational, social and leisure-physical activities. In light of this trend, recent studies have emphasized the importance of using a comprehensive assessment of participation that can capture even small incremental changes in function after acute hospitalization in older adults [5–7].

One of the major factors associated with participation of older adults is cognition [8, 9]. Executive functions in particular play an essential role in all aspects of daily functions and underlie the ability to cope with cognitively challenging activities [10]. Indeed, executive functions strongly relate to better performance of Instrumental Activities of Daily Living (IADL) [11] and maintaining participation in leisure activities [12]. Acute illness hospitalization in older adults may accelerate or trigger cognitive decline including decrease in executive functions [13, 14]. Cognitive impairment in acutely hospitalized older adults is a major risk factor associated with adverse outcomes including, longer Length Of hospital Stay (LOS), early readmissions, functional decline and mortality [15–17]. Specifically, executive dysfunction predicts hospital readmissions and falls risk [18, 19].

Therefore, screening cognition in hospitalized older adults is recommended [20]. To date, traditional cognitive screening tests of global cognition, such as the Mini-Mental State Examination (MMSE) and Pfeiffer’s Short Portable Mental Status Questionnaire (SPMSQ), are commonly described in studies in acute hospital settings[17, 21, 22] along with the Saint Louis University Mental Status Examination (SLUMS), which is becoming more widely used [23]. Such tools measure isolated cognitive functions (e.g., attention, orientation and memory) and have limited ability to identify patients with milder forms of cognitive impairment and decreased executive functions due to marked ceiling effects among high-functioning older adults [24]. Neuropsychological measures (e.g., the Trail Making Test, Stroop test) were designed to assess components of executive functions. However, their use is less stated in acute illness hospitalization studies. A major critique of both the traditional cognitive screening and the neuropsychological measures of executive functions is their limited ecological validity and therefore decreased ability to predict performance of complex daily life activities (e.g., IADL) [25–27].

Functional cognition assessments may address the limitations of the traditional cognitive and neuropsychological assessments [24]. Functional cognition assessments may be defined as standardized observations on cognitive processes during performance of complex daily activities, such as managing medication and preparing a simple meal [24]. Functional cognition is considered a better indication of functioning in real life situations that rely on executive functions [28]. For example, task initiation and completion, sequencing, safety and judgment are scored during daily task performance as central components of executive functions. Performance-based assessments of functional cognition are sensitive to subtle cognitive and executive functions changes even when performance on traditional cognitive screening tests is intact [29]. These assessments discriminated between healthy older adults and those with mild cognitive impairment [30], stroke patients [31] and persons with traumatic brain injury [32]. In addition, these performance-based assessments are associated with IADL performance and predict the need for home care use in community-dwelling older adults [33, 34].

Clinicians have used performance-based assessments of functional cognition in different settings and across diagnostic groups. Their use was found feasible in acute-settings and discriminated executive dysfunctions effectively, however research has focused mainly on acute stroke patients [35, 36] and adults hospitalized for elective orthopedic surgery [34] and their predictive validity has not been studied in people hospitalized due to acute illness. Implementing the concept of functional cognition in acute settings can improve predictive validity of hospital-based cognitive tests to independent community living after discharge, especially in high functioning older adults (e.g., without a diagnosis of dementia or delirium).

Hence, the aim of this study is to explore whether functional cognition screening during acute hospitalization explains additional variance in participation (i.e., IADL, social and leisure activities) of older adults one and three month after discharge, beyond traditional cognitive screening and neuropsychological measure of executive functions after controlling for confounders (e.g., age, length of stay). We hypothesize that functional cognition will have a unique contribution to the explanation of the change in participation level post-acute hospitalization.

## Methods

### Design and participants

This prospective longitudinal study is part of a larger prospective observational study, Hospitalization Process Effects on Mobility Outcomes and Recovery (HoPE-MOR) [37]. Older adults admitted from February 2019 through February 2020 to one of three internal medicine wards in a medical center in northern Israel, were screened for participation in the current study. Patients recruited for the study had an unplanned admission due to a non-disabling, acute medical illness (e.g., pneumonia and acute bronchitis) and met the following inclusion criteria: (1) age 65 years and above, (2) able to speak, read and write Hebrew, (3) able to sign an informed consent form as determined by a health professional, (4) self-reported pre-admission ability to walk with or without personal or assistive device. Exclusion criteria included (1) admission diagnosis of acute or chronic neurologic disorder, (2) acute orthopedic condition (e.g., fractures), (3) diagnosis of dementia (4) delirium, the presence of delirium was evaluated at admission and daily during hospitalization using the 4'A's Test (4AT) [38], (5) need for mechanical ventilation, (6) prescribed isolation, (7) admitted for end-of-life care. A total of 145 patients were approached for participation in the current study, of them 45 declined, leaving 100 eligible participants. Of the100 participants recruited, eight withdrew their consent during hospitalization or one month after discharge, three were transferred to another ward or discharged after less than 24 h, three were discharged to a rehabilitation facility, and two participants were unable to contact at one month, leading to 84 participants that completed one month follow up. In the three months’ follow-up, one participant withdrew consent and one died. In addition, the three months’ follow-up of five participants was during the first outbreak of the COVID-19 pandemic therefore their data were excluded from analysis, leaving 77 participants at three months follow-up.

The sample size was calculated (using G*Power) for linear multiple regression R^2^ increase and the following parameters: significance level of 0.05, power of 0.80, and effect size of f^2^ = 0.15 for four stages (blocks) and a total of five predictors. Based on this calculation, the total number of participants needed for this study was 85. Nevertheless, a rule-of-thumb suggests that 10 subjects per variable is the minimum required sample size for linear regression models to ensure accurate prediction [39], indicating an acceptable sample size for the present study.

### Data collection

Trained research assistants administered the assessment measures and questionnaires during the first 48 h of hospitalization in the internal medicine ward, and after obtaining an informed consent. At one-month post-discharge the follow up was conducted at the participant’s homes and at three months, the follow up was conducted by a telephone interview. The same research assistant (an experienced occupational therapist) administered the measure of participation during hospitalization and after discharge, as well as the functional cognition screening measure during hospitalization.

### Measurements

#### Main outcome-participation

Participation was measured by the Activity Card Sort (ACS) version 1 that is valid in Israel [40]. An interview version was conducted to determine the impact of the acute illness on the individual's level of activity and participation compared with before hospitalization. The ACS consists of 88 daily activities divided into four categories: IADL (e.g., grocery shopping, meal preparation, household care, financial management, driving and using public transportation), social and leisure activities (e.g., meeting with family and friends, attending the cinema, theater and restaurants, participating in religious activities), leisure activities with low physical demands (e.g., watching television, reading, listening to music, painting) and leisure activities with high physical demands (e.g. walking, exercising, swimming, travelling, gardening). During hospitalization, participants were asked whether they participated in each activity before the current hospitalization (no = 0; yes = 1) i.e. baseline participation level. In the follow-up visits after one and three months participants rated the activities as currently doing (same = 1 or less = 0.5) or not doing anymore (= 0). A total “retained activity level” score (0–100) is calculated as a percentage, that is the sum of weighted scores of activities in which a person is currently engaged divided by those they were engaged with before hospitalization. Lower percentages indicate more significant withdrawal from previous participation. The ACS is used worldwide and has been translated, adapted and validated in Israel [41, 42]. The original ACS has a good test–retest reliability (*r* = 0.90) in community dwelling adults [40]. The ACS has a good construct validity, as demonstrated in studies comparing activity levels in different age groups and populations with neurological disabilities [41, 43].

#### Predictors

In-hospital characteristics and risk factors were chosen according to the well-documented predictors of acute hospitalization outcomes described in previous studies [4, 44]. In the present study these factors include cognitive status at admission, emotional status, functional status in BADL during hospitalization, Length of Hospital Stay (LOS), severity of the acute illness, and symptoms severity. Baseline characteristics i.e. before current hospitalization include comorbidities and premorbid functional status. Sociodemographic characteristics include age, gender, years of education and living situation. All measurements were administered at admission to the internal medicine ward. Functional status in BADL was assessed also at discharge.

Basic cognitive status was assessed by the Mini Mental State Examination Telephone version (MMSET) [45]. The original Mini-Mental State Examination (MMSE) [46] is a widely used screening tool for cognition in older, community-dwelling, hospitalized, and institutionalized adults. In the current study we used the 22-point telephone version of the MMSE (MMSET). Total scores for the original face-to face MMSE and telephone versions of the MMSE correlate strongly (Pearson’s *r* = .85, *P* < .001).

Executive functions were assessed using the Color Trails Test (CTT) [47], a neuropsychological paper and pencil test which measures executive skills, sustained and divided attention. The CTT is a variant of the validated Trail Making Test and was developed to minimize cultural and language bias. It consists of two subtests, for the CTT part 1, the respondent uses a pencil to rapidly connect, in sequence, circles numbered 1 through 25. For the CTT part 2, the respondent rapidly connects numbered circles in sequence, but alternates between pink and yellow colors. Scoring is calculated by measuring completion time (up to 240 s). One of the advantages of the CTT is that it is quick to administer. Standardized T scores are derived from normative data correcting for age and years of education.

Functional cognition was measured by the medication management subtest of the alternative version of the Executive Function Performance Test (aEFPT) [48]. The aEFPT is an addition of the valid EFPT [31], a performance-based assessment which measures executive functions while carrying out daily tasks. The subtest used in the current study assess medication management abilities necessary for independent daily function. The task involves sorting medications into a 7-day pill sorter, using 3 prescription bottles. The patient has to ignore distractors (other bottles) and to follow the specific sorting instructions. The examiner evaluates the individual’s executive functioning on five components: task initiation, organization, sequencing of task components, safety and judgment, and task completion. The EFPT uses a standardized hierarchical cueing system that is scored from 0 (no cue required) to 5 (the participant cannot do the task). The score for each executive function component ranges from 0 to 5, and the total score of the task ranges from 0 to 25, such that a higher score indicates the need for more assistance. The medication management task from the original EFPT has good interrater reliability (.87) [31]. The alternate medication management task from the aEFPT is more complex and discriminates between patients with stroke and healthy controls [48].

Emotional status was assessed using the Hospital Anxiety and Depression Scale (HADS), a brief questionnaire containing 14 items [49]. The HADS was originally designed to detect emotional disturbances in non-psychiatric patients, it measures anxiety and depression on two separate scales (seven items for each scale). The scores range from 0 to 3 points for each question, with a maximum score of 21 for each scale. Depressive symptoms were coded as normal (0-7 points), borderline (8-10 points) and positive symptoms of depression (11 points and above) categories.

BADL were measured by the Modified Barthel Index (mBI) [50]. The mBI consists of individual's subjective assessment of their independence level in 10 items of BADL: feeding, bathing, grooming, dressing, bowel and bladder care, toilet use, ambulation, transfers, and stair climbing. The total score ranges from 0 to 100, with lower scores indicating increased disability and more assistance required. Baseline (premorbid) mBI was assessed on admission retrospectively for 2 weeks before admission and at discharge. A functional decline score was calculated and defined as a decrease of 5 points or more on the mBI total score from baseline pre-morbid status to discharge, which express a loss of independence in one of the BADL domains [51].

Severity of symptoms was assessed at admission using 11 self-rated common symptoms (e.g., dyspnea, cough, pain) [52]. Patients rated the presence and intensity of symptoms experienced during the past 24 h on a 0–10 scale with higher scores indicating greater severity.

The following information was obtained from patient's medical records: LOS, comorbidities and severity of the acute illness. Comorbidities were assessed using the Charlson Comorbidity Index (CCI) [53]. The CCI weights the number and severity of 19 health conditions representing patient chronic health status. Weighted scores for each condition range from 1 to 6, resulting in a maximum score of 33, with higher scores indicating greater comorbidity. Severity of illness was measured using the National Early Warning Score (NEWS) [54], assessing the objective severity of the patient's condition by the degree of deviation from the norm (0 to 3) of seven physiological parameters: respiratory rate, oxygen saturation level, supplemental oxygen use, temperature, heart rate, systolic blood pressure, and conscious state resulting in a maximum score of 21, with higher scores indicating greater severity.

### Statistical analysis

Descriptive data were presented as frequencies for categorical variables and mean and standard deviation for continuous variables. A log10 transformation was performed to normalize the left skewed distribution of the participation outcome ACS total retained score. After transformation, distribution became normal and the direction of the scale has changed indicating that higher numbers represent lower participation. Transformation of the mBI data did not correct the non-normal distribution. All other study variables were normally distributed. Independent sample t-test and Mann–Whitney tests for continuous variables and chi-squared tests for categorical variables were used as sensitivity analysis to test for significant differences in study variables between participants that completed the study versus those who withdrew. A bivariate Pearson correlation analysis (Spearman for the mBI) was performed to examine relationships between study variables with participation and between the independent variables. A hierarchical linear regression model was used to examine whether the functional-cognitive screening explains additional variance in participation beyond other cognitive measures while controlling for confounders. The ACS total retained score was used as the dependent variable and independent variables were entered in blocks according to the bivariate correlations. For all analyses significance was accepted at level p ≤ 0.05. All analyses were performed using SPSS version 25 statistical program (SPSS Inc., Chicago, IL).

## Results

Table 1 presents characteristics of the study sample. The participants had a mean age of 76.6 years (SD ± 7.4, range 65–94), and 53% (41) were female. Pneumonia, congestive heart failure, and chronic obstructive pulmonary disease were the most common diagnoses on admission. Sensitivity analysis revealed no significant differences (*p* > 0.005) between the 77 participants that completed the three months follow-up and the 23 who withdrew (including comparison between seven participants that completed only the one month follow-up), in all basic characteristics (age, gender, years of education, LOS, mBI, illness severity, and comorbidity). Most of the participants were independent in BADL (mBI above 80) two weeks before admission (94%) and at discharge (91%), only 13% of the participants experienced functional decline from pre-morbid stage to discharge.

**Table 1:** Sample characteristics

Characteristic	After 1 month (*n* = 84) Mean (SD) Range	After 3 months (*n* = 77) Mean (SD) Range
Gender (females), n (%)	45 (54)	41 (53)
Age, years	76.6 (7.3) 65–94	76.6 (7.4) 65–94
Education, years	12.5 (4.3) 3–25	12.4 (4.3) 3–25
Living alone, n (%)	30 (35)	26 (34)
Pre-morbid mBI score (0–100) [median]	96.7 (7.4) [100] 65–100	96.3 (8.1) [100] 65–100
mBI score at discharge (0–100) [median]	94.4 (12) [100] 24–100	94.1 (12.4) [100] 24–100
Length of hospital stay in days	5.7 (4.4) 2–25	5.8 (4.5) 2–25
Illness severity (NEWS score, 0–21)	2.1 (1.9) 0–7	2.1 (1.9) 0–7
Symptoms severity at admission (0–10)	2.6 (1.6) 0–7.2	2.7 (1.6) 0–7.2
Comorbidity (CCI score, 0–33)	1.5 (1.6) 0–6	1.6 (1.6) 0–6
MMSET Score at admission (0–22)	18.7 (2.7) 11–22	18.8 (2.6) 11–22
Color Trails Test- part 1 at admission, T score	32.5 (12.1) 20–62	33.1 (12.3) 20–62
Color Trails Test- part 2, at admission T score	32.1 (11.2) 20–59	32.1 (11.4) 20–59
Med-aEFPT at admission (0–25)	5 (4.1) 0–16	5.1 (3.9) 0–16
HADS total score at admission	13.3 (4.6) 6–27	13.1 (4.4) 6–27
Depressive symptoms at admission (HADS score 11 +), n (%)	15 (18)	15 (19)

*Abbreviations*: *mBI* Modified Barthel Index, *NEWS* National Early Warning Score, *CCI* Charlson Comorbidity Index, *MMSET* Mini-Mental State Examination Telephone version, *Med-aEFPT* Medication sorting task from the alternative Executive Functions Performance Test, *HADS* Hospital Anxiety and Depression Scale

*Note.* Due to different sample sizes, sample characteristics are included for each follow-up time point

As for participation in daily life (dependent variable), according to the ACS, the mean retained activity level was 84.7% (SD = 17.8, range 36–100) after one month and 87.8% (SD = 16.4, range 46–100) after three months. Table 2 displays bivariate correlations among study variables with participation. After one month, age, gender, LOS, CTT part two and the medication sorting task from the aEFPT were significantly correlated with the ACS retained score and were entered as independent variables in the regression analysis. After three months only age and the medication sorting task were correlated with the outcome. Correlations analysis among the independent variables are described in Table 3. Two hierarchical linear regression models were conducted with participation as the dependent variable (in the first model participation after one month and in the second model participation after three months). For consistency, each included four blocks: age and gender were entered into block one, LOS was entered into block two, the CTT part two was entered into block three and the medication sorting task was added to block four. After one month, the overall model explained 28.4% of the variance in the ACS retained score. Age and gender, explained 18.6% of the variance. LOS and CTT part two were not significant contributors to the change in ACS retained score. The medication sorting task explained additional 5.5% of the variance of participation (Table 4). After three months, the overall model explained 19.5% of the variance in the ACS retained score. Age and gender, explained 13.5% of the variance. LOS and CTT part two were not significant contributors to the change in ACS retained scores. The medication sorting task from the aEFPT explained additional 5.1% of the variance (Table 5).

**Table 2:** Bivariate correlations among study variables with participation (ACS)

	ACS after 1 month (*n* = 84), r(p)	ACS after 3 month (*n* = 77), r(p)
Age, years	.336** .002	.316** .005
Gender	.245* .025	.169 .141
Education, years	-.046 .675	-1.25 .280
Living alone	.157 .173	-.002 .998
Decline in mBI pre-morbid to discharge	-.114 .301	-.157 .172
Length of hospital stay in days	.248** .023	.108 .350
Illness severity (NEWS)	.135 .222	-.034 .770
Symptoms severity at admission	.159 .154	.181 .121
Comorbidity (CCI score)	.049 .659	.124 .284
MMSET Score at admission	-.007 .948	.057 .623
Color Trails Test-part 1 at admission	-.142 .204	-.111 .234
Color Trails Test-part 2 at admission	-.247* .024	.186 .105
Med- aEFPT at admission	.395*** .000	.360** .001
Emotional status (HADS) at admission	-.029 .794	-.043 .714

*Abbreviations*: *mBI* Modified Barthel Index, *NEWS* National Early Warning Score, *CCI* Charlson Comorbidity Index, *MMSET* Mini-Mental State Examination Telephone version, *Med-aEFPT* Medication sorting task from the alternative Executive Functions Performance Test, *HADS* Hospital Anxiety and Depression Scale

*Note*. *P < 0.05, **P < 0.01, ***P < 0.001

**Table 3:** Correlations between independent variables entered into the regression analysis

**After one month (*n*** = **84)**					
	Age	Gender	LOS	CTT-2	Med-aEFPT
Age	1				
Gender	-.070	1			
LOS	.328**	-.089	1		
CTT-2	-.314**	-.014	-.161	1	
Med-aEFPT	.381***	-.071	.061	-.333**	1
**After three months** **(*n*** = **77)**					
	Age	Gender	LOS	CTT-2	Med-aEFPT
Age	1				
Gender	-.062	1			
LOS	.338**	-.114	1		
CTT-2	-.321**	.039	-.155	1	
Med-aEFPT	.386**	.084	.029	-.332**	1

*Abbreviations*: *LOS* Length of hospital Stay, *CTT-2* Color Trail Test part 2, *Med-aEFPT* Medication sorting task from the alternative Executive Functions Performance Test

*Note*. *P < 0.05, **P < 0.01, ***P < 0.001** s**

**Table 4:** Hierarchical multiple regression for change in participation level after one month (*n* = 84)

	B	SE B	β (p)	R^2^	ΔR^2^	F(p)
**Block 1**						
Age	.031	.009	.355	.186	.186	9.22 (.000)
Gender	.341	.127	.270			
**Block 2**						
LOS	.025	.015	.173	.212	.027	2.71 (.104)
**Block 3**						
CTT part 2	-.008	.006	-.135	.229	.016	1.67 (.199)
**Block 4**						
Med- aEFPT	.042	.017	.265	.284	.055	5.99 (.017)

*Abbreviations*: *LOS* Length of hospital Stay, *CTT* Color Trail Test, *Med-aEFPT* Medication sorting task from the alternative Executive Functions Performance Test

**Table 5:** Hierarchical multiple regression for change in participation level after three months (*n* = 77)

	B	SE B	β (p)	R^2^	ΔR^2^	F(p)
**Block 1**						
Age	.027	.009	.327	.135	.135	5.79 (.005)
Gender	.231	.132	.190			
**Block 2**						
LOS	.003	.016	.022	.136	.000	.035(.852)
**Block 3**						
CTT part 2	-.005	.006	-.098	.144	.009	.719 (.399)
**Block 4**						
Med- aEFPT	.039	.019	.255	.195	.051	4.48 (.038)

*Abbreviations*: *LOS* Length of hospital Stay, *CTT* Color Trail Test, *Med-aEFPT* Medication sorting task from the alternative Executive Functions Performance Test

## Discussion

This study aimed to examine the contribution of functional cognition during acute illness hospitalization as a predictor of post- hospitalization participation. Consistent with our hypothesis functional cognition screening during acute hospitalization had significant unique contribution to the explanation of the change in participation level after one and three months post-discharge. This main finding is in line with previous studies demonstrating that functional cognition strongly relates to IADL and independent community living in healthy community dwelling older adults and those with mild cognitive impairment and dementia [33, 55, 56]. Although the contribution of functional cognition in this study was relatively small, it outperformed the other cognitive measures in predicting participation declines. The traditional cognitive screening tool (i.e., MMSET) was not significantly correlated with participation. The neuropsychological measure (i.e., CTT) detected executive dysfunctions, however it did not have a significant contribution to the explanation of participation post discharge. These findings strengthen the importance of using ecologically valid cognitive measures. Although the use of validated assessments of functional cognition is valuable in many settings, this is the first study to demonstrate the importance of functional cognition screening during acute illness hospitalization in detecting subtle cognitive deficits and its potential in predicting participation in a wide range of life domains after discharge.

The current sample consisted of relatively high-functioning older adults, mostly independent in basic functions before and after hospitalization, without a diagnosis of dementia or delirium. They can be viewed as an example of a sub-sample of hospitalized older adults that may be going undetected by the traditional cognitive tests such as the MMSE and by the common outcome of functional decline (i.e., BADL). Basic activities mainly rely on physical abilities and procedural memory and therefore are performed routinely and automatically. However, participation in complex daily activities such as IADL and social activities requires higher cognitive skills (i.e., executive functions) [10]. As we shown in the current study, older adults with executive dysfunction can be completely independent in BADL, yet have difficulties resuming participation in IADL, leisure, and social activities due to greater cognitive demands, consequently affecting their community reintegration after hospitalization. Performance-based assessments of functional cognition include scoring of crucial components of executive functions relevant to independent community living such as safety and judgment that are overlooked by the traditional cognitive and neuropsychological assessments. Hence, the results of the current study support the uniqueness of functional cognition as a construct and are in line with the growing support of it's importance among researchers [24, 26].

To date, there is scarce report and no agreed upon screening measure for functional cognition in acute settings. Due to restricted time and resources to assess patients prior to discharge, a short and easy to administer (< 5 min), bedside screening assessment for functional cognition with limited equipment, indicative of executive deficits across different diagnostic groups and predictive of IADL performance is recommended [24]. In the current study, we screened for functional cognition by using a standardized task of medication management ability which is highly relevant during acute and chronic illness and is recommended as part of global geriatric assessment [57]. Medication management is an essential daily activity that requires executive functions skills. Indeed, older adults even with mild executive dysfunction are at an increased risk for mismanaging their medication [58]. Poor medication self-management skills in community dwelling older adults are associated with functional decline, and increased healthcare utilization, including emergency department visits [59, 60].

The results of this study are in line with previous studies supporting the use of functional cognition screening measures during hospitalization and in the community. For example, Edwards et al. [34] examined the use of a relatively new performance-based screening measure of functional cognition, the menu task, among community dwelling adults and patients hospitalized for orthopedic surgery. Participants classified as impaired on the menu task performed significantly lower on the neuropsychological screening tests and IADL measures. Marks et al. [55]examined the use of the Medi-Cog-Revised, a combination measure of the mini-cog and the medication transfer screen revised. The combined functional-cognition screening measure identified the potential for IADL dependency among community-dwelling adults (> 55 years) better than either measure alone. Overall, it is recommended to have a few screening measures of functional cognition to choose from, according to the specific needs of each patient in addition to the traditional cognitive screening measures. Incorporating functional cognition, using performance-based screening tests, into acute-settings may help identify older adults in need of more comprehensive assessment and may guide the planning of appropriate post-discharge services. Older adults who successfully complete the traditional cognitive screening tests during hospitalization (e.g., MMSE), but with impaired performance on a functional cognition screening test could be referred to occupational therapy for further evaluation and intervention in the community, to prevent participation declines. The medication management task from the aEFPT used in the current study may be a useful screening measure for functional cognition.

### Study limitations

The first limitation relates to the homogenous sample recruited from a single site which limits the generalizability of the results. The limitation of the medication sorting subtest used in the current study is the need for equipment. Despite that, the test is easy to use and can be administered bedside with lightweight portable equipment that best simulates real-life medication sorting. In addition, the ACS is a comprehensive measure of participation and as such, it takes a relatively long time to administer. The use of yes/no responses in the ACS does not give insight into how often or how regularly individuals participate in these activities before their hospitalization. Furthermore, the ACS is a self-report measure with retrospective recall. Limitations of self-report measures administered at different time points include the potential for recall bias and reliance on patients' subjective estimation of their level of participation in activities. To reduce the length of assessment in acute settings, it is recommended to integrate brief questions that focus on patients’ unique characteristics of participation beyond BADL and to administer a comprehensive assessment of participation during a follow-up visit after discharge. Future studies should examine the use of other patient-centered measures to reflect participation, such as the Canadian Occupational Performance Measure (COPM). The overall explained variance of participation after one and three months was relatively low. This finding suggests that other factors may explain participation that have not been examined in this study, such as specific morbidities, socioeconomic status measured only by education and not income, social support, and mobility (e.g., gait speed) during hospitalization. Future studies should explore the generalization of the current findings to more vulnerable populations, such as frail older adults and adults suffering from brain injuries. In addition, future research should examine participation recovery and the contribution of functional cognition among patients with specific acute illness diagnoses and morbidities.

## Conclusions

By broadening and refining the assessment of cognition currently used in acute settings to include performance-based evaluation of functional cognition, healthcare professionals would be able to better detect older adults with mild executive dysfunctions, plan the most effective interventions and ensure patients are best prepared to re-integrate into their community following discharge. Administration of a short screening measure of functional cognition is recommended in acute settings, especially for high functioning older adults that perform well on traditional cognitive screening tests. This will enable to better predict participation in daily life after discharge and possibly lead to decreased hospital readmissions and reduced healthcare utilization. More studies investigating the role of functional cognition assessment during acute settings with diverse patient populations, using different screening measures are recommended.

## Data Availability

The datasets used and/or analyzed during the current study are available on reasonable request from the corresponding author.

## Abbreviations

BADL: Basic Activities of Daily Living
IADL: Instrumental Activities of Daily Living
LOS: Length Of hospital Stay
ACS: Activity Card Sort
mBI: Modified Barthel Index
NEWS: National Early Warning Score
CCI: Charlson Comorbidity Index
4AT: 4’A’s Test
MMSE: Mini-Mental State Examination
MMSET: Mini-Mental State Examination Telephone version
aEFPT: Alternative Executive Function Performance Test
CTT: Color Trails Test
HADS: Hospital Anxiety and Depression Scale
